# Transactions of the American Gynæcological Society for 1878

**Published:** 1879-07

**Authors:** 


TRANSACTIONS OF THE AMERICAN GYNÆOLOGICAL SOCIETY FOR THE YEAR 1878. Vol. III; Houghton, Osgood & Co. Boston.

With this volume a new style of binding has been adopted which is both neater and more durable. Fellows and subscribers desiring the back volumes of uniform binding with this, can, at the cost of fifty cents additional, have such furnished.

We find in the present volume many very interesting and important papers. Dr. H. F. Campbell presented an essay on "Rectal alimentation in the nausea and inanition of pregnancy, in which intestinal inhaustion was considered as an important factor, and the true solution of its efficiency." Many instructive data are given, and cases reported demonstrative of the theories involved. "The Transaction" is a very desirable book, and should be in every library.

